# The Role of Radial Shockwave Therapy in Reducing Spasticity and Improving Upper-Limb Function Following Stroke

**DOI:** 10.3390/jcm15155879

**Published:** 2026-07-28

**Authors:** Klaudia Marek, Joanna Piwnik, Elżbieta Miller

**Affiliations:** 1 Department of Neurological Rehabilitation, Medical University of Lodz, Milionowa 14, 93-113 Lodz, Poland; elzbieta.dorota.miller@umed.lodz.pl; 2 Department of Biostatistics and Translational Medicine, Medical University of Lodz, Mazowiecka 15, 92-215 Lodz, Poland; joanna.piwnik@umed.lodz.pl

**Keywords:** spasticity, stroke, radial shockwave

## Abstract

**Background**: Spasticity resulting from a stroke usually develops within a few weeks or months of the incident and occurs in approximately 43 percent of patients. Recently, noninvasive radial shockwave therapy has been gaining increasing attention as a treatment for spastic muscles, leading to a decline in muscle tone. This study aims to evaluate the effectiveness of rESWT in reducing muscle tone and improving hand function in stroke patients with upper-limb spasticity. **Methods**: We conducted a study involving 62 stroke patients with upper-limb spasticity (MAS ≥ 2). The ESWT group received four sessions of radial shockwave therapy applied to the wrist flexor muscles (flexor carpi radialis, flexor carpi ulnaris, palmaris longus, pronator teres, pronator quadratus, and flexor digitorum superficialis) at 6-day intervals (3500 pulses, 8 Hz, 2.5 bar), combined with standard rehabilitation during hospitalization. The non-ESWT group followed a standard rehabilitation program. Assessments were performed using the following scales: FMA-UE, ADL, MAS, radiocarpal joint extension range of motion, and VAS. Measurements were obtained at three time points: before the intervention, one week after the first intervention, and one week after the fourth intervention. **Results**: The ESWT group showed improvements in upper-limb motor function (FMA-UE, *p* < 0.0001) and functional independence (ADL, *p* < 0.0001). They also experienced an increase in radiocarpal joint range of motion (ROM, *p* > 0.0001) and a reduction in spasticity (MAS, *p* < 0.0001). **Conclusions**: The intervention involving four ESWT sessions combined with conventional rehabilitation results in improved hand motor function, reduced spasticity, increased extension of the radiocarpal joint, and increased functional independence. Improvements in upper-limb function and reductions in spasticity may occur independently of changes in pain perception.

## 1. Introduction

Spasticity is a prevalent complication resulting from stroke, which impedes the recovery of functional ability and affects up to 43 percent of stroke survivors [1]. The progression of spasticity depends on the patient’s clinical presentation. However, it most commonly occurs within the first three months following a stroke. Studies by Wissel et al. (2015) indicated that in nearly half of patients with motor impairments, spasticity begins within the first 10 days following a stroke [2,3]. It is a form of muscle hypertonia resulting from damage to the pyramidal tract, specifically the corticospinal fibers of the extrapyramidal tract. Spasticity is a hallmark of positive upper motor neuron (UMN) syndrome resulting from central nervous system (CNS) damage [4]. First described by Lance in 1980, muscle hypertonia is characterized by an increase in tonic stretch reflexes that depend on speed and result in exaggerated tendon jerks. This increase is caused by excessive excitability of stretch reflexes [5]. The term “muscle hypertonia” refers to a common clinical phenomenon involving increased joint resistance to passive stretching. Research in this area suggests that hypertonia may have various neural and non-neural components, whereas spasticity is primarily neural in nature [6]. Three weeks post-stroke, the muscle undergoes significant structural and rheological changes (physical property changes) as spasticity begins to develop, usually within the first 1 to 6 weeks. While initially driven by neural changes (increased excitability of the stretch reflex), these changes are quickly followed by alterations in the muscle tissue itself, including increased stiffness, reduced excitability, and architectural changes [7,8].

As neurology and rehabilitation have advanced, the definition of spasticity has evolved. Pandyan et al. (2005) introduced the term “impaired sensorimotor control resulting from upper motor neuron damage and manifested by periodic or prolonged involuntary muscle activation” [9]. This refined definition was adopted by the Support Program for the Assembly of a Database for Spasticity Measurement (SPASM) as the most current. This definition considers the influence of viscoelastic soft tissue properties on joint stiffness and the role of proprioceptive and cutaneous sensory pathways in selecting effective treatments and understanding pathophysiology [10].

Motor disorders like spasticity are clinical symptoms that can be explained by abnormal neuroplasticity processes. After a stroke, disturbances in this process manifest in various aspects of motor behavior in subacute and chronic conditions, including abnormal muscle strength control, abnormal muscle activation, synergistic muscle activation, limb coupling, and general neuromechanical consequences [11]. Patients with spasticity may suffer from chronic neuropathic pain, sensory disturbances, severe muscle spasms, skeletal deformities, muscle fibrosis, and muscle atrophy [12]. Additionally, patients experience non-reflex hypertonia due to changes in connective tissue [7]. In chronic spasticity, a decrease in the number of sarcomeres occurs, resulting in an increased proportion of connective tissue in the affected muscles [13]. Persistent hypertonia results from prolonged muscle contraction around blood vessels, leading to local hypoxia and the development of pain. Chronic inflammation activates nociceptive receptors in the muscles [14]. A 2026 meta-analysis by Tamayo et al. showed 65 percent of patients with spasticity reported pain, with 60 percent describing it as throbbing [15].

The most common pattern in patients with upper-limb spasticity is internal rotation of the shoulder joint, adduction and flexion of the elbow and wrist joints, and sometimes flexion of the fingers as well [16]. The relationship between paresis and spasticity is complex and subject to constant change throughout the neurorehabilitation process. A limited range of motion in the wrist joints of stroke patients is associated with increased spasticity, which exacerbates this limitation further. This creates a vicious cycle of deteriorating mobility, resulting in a poorer response to treatment and slower functional improvement [16]. Early treatment for patients with spasticity may improve prognosis by maintaining or improving motor function and reducing secondary musculoskeletal complications [17].

Despite the wide availability of pharmacotherapies, including botulinum toxin injections, phenol neurolysis, epidural therapy, physiotherapy methods, ultrasound and electrostimulation physical therapy, and surgery, spasticity remains common among neurological patients [18,19]. A shockwave is a type of acoustic wave with mechanical properties that can cause significant changes in physical properties. Compression waves are characterized by high amplitude and rapid pressure changes, followed by high- and low-pressure phases. The radial type of shockwave (rESWT) used in physiotherapy has a low peak pressure (0.1 MPa), a long rise time (50 μs), and a duration ranging from 200 to 2000 μs [20,21]. Two physical effects of rESWT can be distinguished: the direct mechanical effect on the treatment site (primary effect) and the indirect mechanical effect resulting from cavitation (secondary effect) [22]. Tissue penetration has a limited depth of action, amounting to approximately 3–4 cm. Compared to focused waves, rESWT treatment is better tolerated by patients [23], and there is a lower risk of skin and soft tissue damage. This is confirmed by the low incidence of complications after therapy [24]. Shockwave therapy (ESWT) is a noninvasive, reversible treatment method. It has been successfully used in orthopedics [25], urology, and lithotripsy [26]. Over the past 10 years, positive results have been published on using ESWT to treat spasticity. This treatment improves motor function, reduces pain, and enhances functional independence, even after a single session [27,28]. A 2022 meta-analysis of 34 ESWT intervention studies confirmed the method’s efficacy and safety in treating spasticity. The analysis demonstrated a reduction in Modified Ashworth Scale (MAS) scores and an increase in passive range of motion in the joints [29]. Intensive neurorehabilitation and occupational therapy have been demonstrated to be highly effective forms of support for recovery after a stroke, as evidenced by substantial scientific documentation [30]. Research has demonstrated that systematic rehabilitation can lead to functional improvement even many years after the incident, with the most significant changes being observed in the initial months. The degree of motor function achieved by patients five years after a stroke is comparable to that observed around the second month of therapy, provided that it is consistently maintained [31]. The necessity of exploring novel treatment strategies and the development of contemporary training modalities, encompassing complementary methods such as rESWT [32], is paramount. In the treatment of spasticity following a stroke, the development of a step-by-step therapy strategy is considered standard practice. Suputtitada et al. (2024) [33] underscore the necessity of integrating complementary methodologies into prevailing spasticity treatment protocols. One such therapeutic modality is rESWT, a technique that has been demonstrated to reduce spasticity for up to 12 weeks [33].

Therefore, the aim of this study is to evaluate the efficacy of rESWT in reducing muscle tone and pain and in improving hand function and functional independence in stroke patients with forearm and hand spasticity, as assessed by the MAS at ≥2, as a potential method that could complement the standard treatment protocol for spasticity.

To verify the study assumptions, one main (primary, H1) hypothesis and three secondary hypotheses were formulated. The main hypothesis posited that in stroke patients with upper-limb spasticity rated at ≥2 points on the MAS, the use of four sessions of rESWT as an adjunct to standard rehabilitation leads to a significant reduction in spasticity compared to standard rehabilitation.

Three separate secondary hypotheses posited that the use of rESWT as an adjunct to standard rehabilitation:

H2: leads to a significantly greater improvement in upper-limb motor function as assessed by the FMA-UE scale.

H3: leads to a significantly greater improvement in independence in performing activities of daily living as assessed by the ADL scale.

H4: leads to a significantly greater reduction in pain intensity as assessed by the VAS compared to standard rehabilitation.

## 2. Materials and Methods

### 2.1. Study Design

The study was conducted at the Department of Neurological Rehabilitation of Dr. Karol Jonscher Municipal Medical Center from May 2023 to April 2025. The study was approved by the Bioethics Committee of the Medical University of Łódź (Poland, RNN/78/23/KE, 18 April 2023). The study was carried out in accordance with the principles of the Declaration of Helsinki. A total of 62 stroke patients (19 women and 43 men) who met the study participation criteria were recruited. Before the study began, all participants signed an informed consent form and were informed that they could withdraw from the study at any time without consequence.

The inclusion criteria for the study were as follows: age greater than or equal to 18 years; ischemic or hemorrhagic stroke; spasticity, as measured by the MAS, of at least 2 points; and signing an informed consent form. The exclusion criteria were: central nervous system disease other than stroke; paralysis or paresis due to causes other than stroke; wrist injuries or diseases; inflammatory diseases accompanied by fever; severe general condition; systemic diseases; severe cardiovascular diseases; conditions after a heart attack within 30 days; unstable angina pectoris; circulatory failure; severe arrhythmia; uncontrolled metabolic and endocrine diseases; respiratory failure; an MMSE score (Mini-Mental State Examination) below 18; spasticity according to the MAS of at least 2 points; and contraindications for shockwave therapy (e.g., a pacemaker, cancer, skin pathology, or coagulopathy). During the initial recruitment phase, eight potential participants were excluded due to meeting the exclusion criteria.

### 2.2. Sample Size Calculation

The G Power analytical software version 3.1.9.2 (University of Kiel, Kiel, Germany) was used to calculate the sample size. A difference between the mean repeatable measures of 20 units was assumed (Mi_1_ = 60; Mi_2_ = 80), with a standard deviation of 15 units for both measurements and a correlation between measurement pairs of r = 0.10. On this basis, a standard error of the mean difference of 20.12 and a standard effect of 0.9938 were calculated. The analysis was performed assuming a Type I error probability of α = 0.05 and a statistical power of 0.80. According to the calculations, the minimum required sample size was *N* = 11 participants.

### 2.3. Participants

**Figure 1:**
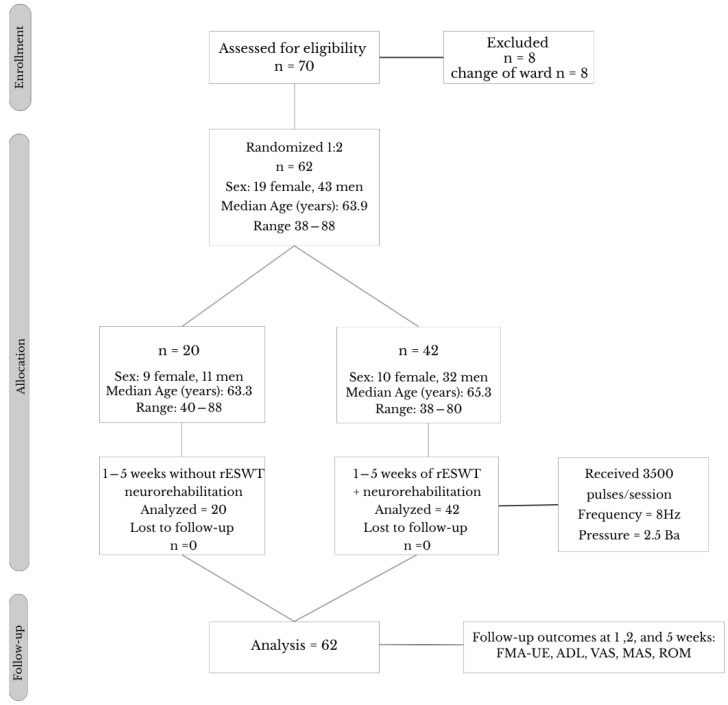
Flowchart of the research. Abbreviations: ESWT: extracorporeal shockwave therapy; non-ESWT: without extracorporeal shockwave therapy; FMA-UE: the Fugl–Meyer Assessment of Upper Extremity; ADL: activities of daily living; VAS: Visual Analog Scale; MAS: Modified Ashworth Scale; ROM: range of motion in the radiocarpal joint (extension).

The study included patients who had experienced an ischemic or hemorrhagic stroke, and their hand spasticity was measured at a level of ≥2 points on the MAS. A total of 62 patients were recruited, comprising 19 women and 43 men with an average age of 64 years and an age range of 38–88 years (Figure 1). A total of 62 patients fulfilled all the criteria for participation in the study. Randomization was performed after the baseline assessment, using a random sequence generated by Random.org. The generated randomization list was recorded and stored in Microsoft Excel. A 1:2 randomization ratio was used (non-ESWT:ESWT group). The assignment was not blinded. Due to the nature of the intervention, it was not possible to conduct a blinded study for either participants or physiotherapists, as ESWT requires the use of specialized equipment and direct contact between the therapist and the patient, which made it impossible to conceal the type of treatment being administered. Patient allocation was performed by a physician according to the predefined randomization sequence, while all clinical assessments and therapeutic procedures were conducted by a physiotherapist.

Patients in the ESWT group and the non-ESWT group participated in an identical, standard rehabilitation program throughout their hospital stay. The program included individualized physical therapy using neurophysiological methods (PNF, NDT-Bobath); task-oriented functional upper-limb training; passive, active–passive, and active exercises through the full range of motion; stretching and strengthening exercises; and balance, coordination, and gait training. In addition, occupational therapy aimed at improving the performance of activities of daily living, speech therapy, psychological support, nursing care, and medical care were provided. Each patient participated in a 60 min individual physical therapy session once a day, five days a week, as well as a 30 min aerobic training session supervised by a physical therapist and a 15 min psychological session. In clinically justified cases, additional physical therapy treatments were also administered in accordance with the attending physician’s recommendations. The duration and intensity of therapy could be adjusted based on the patient’s clinical condition and functional progress. Aside from the rESWT intervention, both groups received the same rehabilitation program. The ESWT group received four ESWT interventions, each six days apart, applied to the group of wrist flexor muscles (flexor carpi radialis, flexor carpi ulnaris, palmaris longus, pronator teres, pronator quadratus, and flexor digitorum superficialis). The precise flowchart of research is presented below (Figure 1).

In the enrollment stage, 70 post-stroke patients with hand spasticity who were admitted to the neurological rehabilitation ward and met the study inclusion criteria were evaluated. Before the first treatment could begin, 8 patients had to be transferred to another ward due to a deterioration in their health resulting from post-stroke complications. Randomization included 62 patients, who were then allocated to the ESWT and non-ESWT groups. The entire follow-up process was completed with full participation by all 62 patients, who were included in the main analysis.

### 2.4. Radial Shockwave Therapy

**Figure 2:**
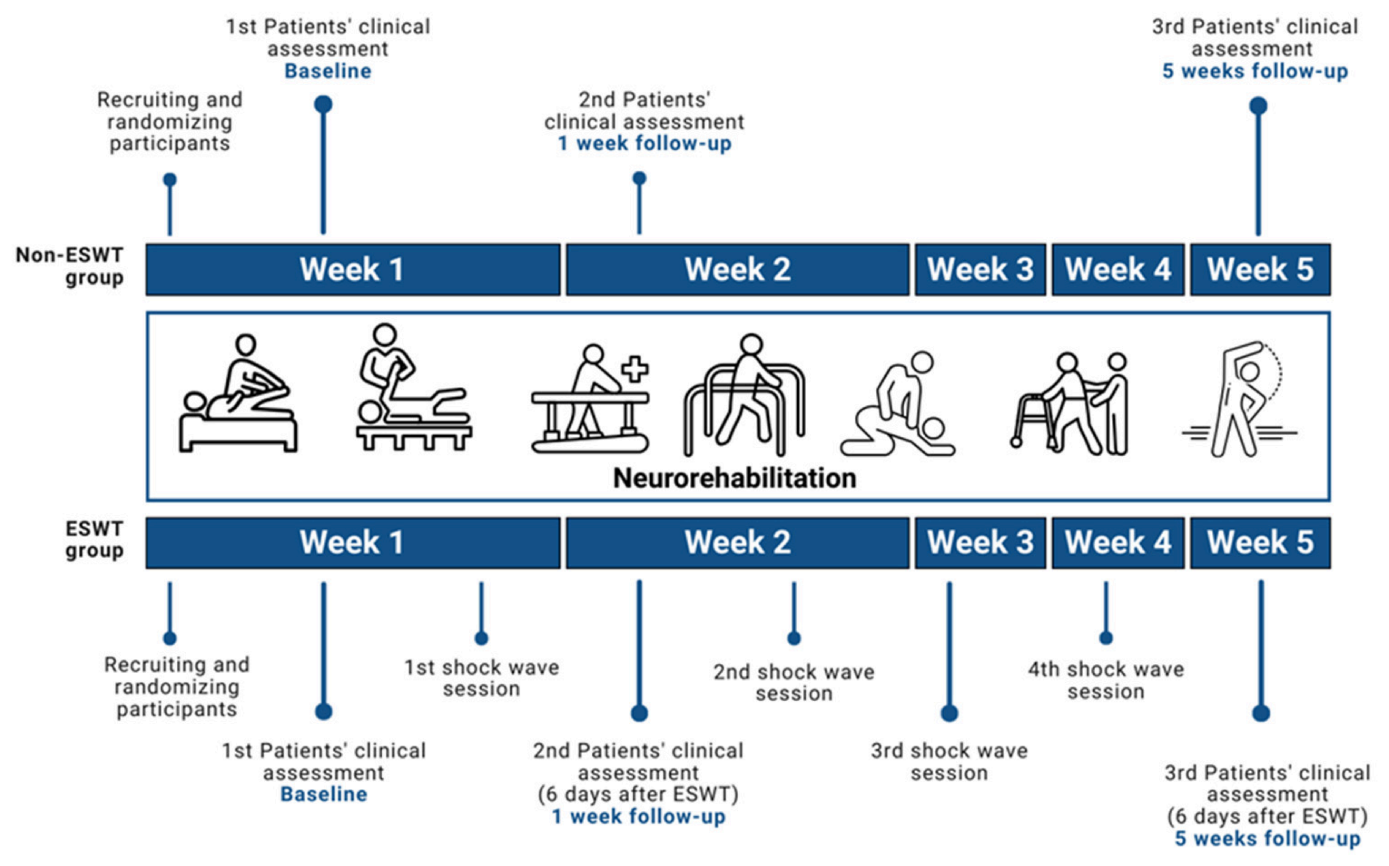
Study timeline and details.

Patients in the ESWT group received four radial shockwave treatments to the spastic flexor muscles in the forearm area. One rESWT session was held once a week, every six days. The following parameters were used: frequency = 8 Hz, pressure = 2.5 Ba, emission mode—continuous, titanium transmitter—15 cm, and number of impacts—3.500. The treatments were performed using the IMPACTIS M+ shockwave device (Astar, Bielsko-Biała, Poland). The procedure did not require anesthesia or pain medication. Assessments were performed before the first rESWT treatment, before the second treatment, and 6 days after the fourth treatment. In total, each patient involved in the study was examined 3 times (Figure 2). In the case of high pain levels, the team was prepared to halt the procedure if necessary.

**Figure 3:**
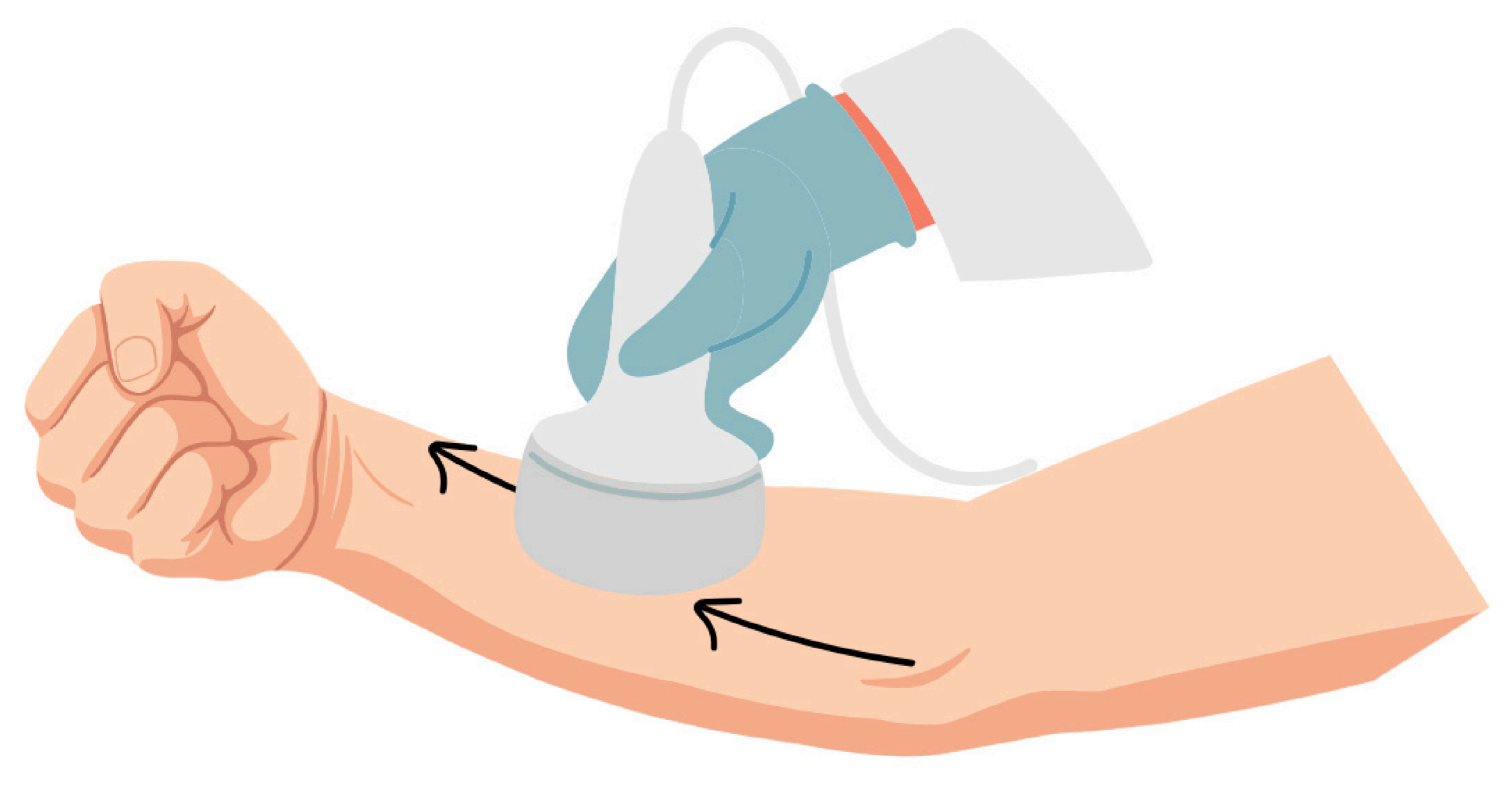
Illustration of the procedure and the depth of rESWT penetration.

The procedure was performed on the anterior (palmar) surface of the forearm, within the wrist flexor group, which includes the flexor carpi radialis, the flexor carpi ulnaris, the palmaris longus, the pronator teres, the pronator quadratus, and the flexor digitorum superficialis. The duration of each therapy session was determined by a predetermined number of pulses (3500 pulses per session) and lasted approximately 5 min. The therapy head was moved continuously and dynamically within the treatment area, without any interruptions during application. The application was performed longitudinally, along the axis of the forearm, following the course of the muscle fibers (Figure 3).

The study design was adapted to the length of patients’ hospitalization, which, in accordance with current guidelines, includes a six-week stay in a neurological rehabilitation ward. For this project, we opted for four weekly ESWT treatments for stroke patients, in line with the approach taken by Brunelli et al. (2022) and Özbek et al. (2024) [34,35]. A systematic review by Starosta et al. (2024) [36] analyzed 16 clinical studies in which ESWT interventions were used to treat hand spasticity in stroke patients. The shockwave parameters ranged from 1000 to 6000 impacts, but most studies used a maximum of 3500 impacts. The pressure was between 1 and 3.5 bar, and the frequency was between 4 and 18 Hz. Therefore, we decided to administer 3500 impacts to the wrist flexor muscles at a frequency of 8 Hz and a pressure of 2,5 bar. These parameters were inspired by a study by Guo et al. (2019) [37], which produced statistically significant results in terms of spasticity reduction (MAS) and hand function improvement (FMA-UE) at each of the five time points of the study (0/1/3/6/12 months) for the group that received ESWT treatments alongside hand mirror therapy. The group receiving only ESWT, without additional rehabilitation, obtained statistically significant results at 3, 6 and 12 months in terms of hand function improvement and spasticity reduction. Radial shockwave therapy was used in most of the studies on the use of ESWT that have been described so far. In terms of treatment volume, the study by Savevska et al. (2016) deserves special attention, as it was one of the few to use 3500 pulses during a single rESWT session for the treatment of upper-limb spasticity [38]. These parameters were used as a reference point in developing our treatment protocol. We decided to use a higher number of pulses because the duration of the procedure did not pose a significant organizational constraint, and the increase in session length associated with a higher number of pulses was minimal. Consequently, we decided on a protocol involving 3500 pulses per session.

Patients were evaluated three times at predetermined time points. The first evaluation took place one week into the study, before the first treatment (baseline). The second assessment took place six days after the first treatment (1-week follow-up), just before the second ESWT session. The final assessment took place six days after the fourth ESWT treatment (5-week follow-up), in the fifth week of the study. Each participant underwent a total of three full assessments: before the intervention, in the early post-treatment phase following the first session, and after completing the entire treatment period.

The following performance measures were:

- Fugl–Meyer Assessment of Motor Recovery after Stroke (FMA-UE);
- Modified Katz Activities of Daily Living Scale (ADL);
- Visual Analog Scale for Wrist Pain (VAS Wrist);
- Visual Analog Scale during ESWT (VAS ESWT);
- Range of motion in the radiocarpal joint (ROM extension);
- Modified Ashworth Scale (MAS).

The Modified Ashworth Scale (MAS) was used to evaluate muscle tone and spasticity. The MAS is a widely used tool for evaluating increased muscle tone in patients with neurological conditions. It is a popular choice among clinicians due to its speed and ease of use. It does not require advanced training to use, making it easy to master even for novice professionals [39]. The traditional Ashworth scale was a 5-point numerical scale that assessed spasticity from 0 to 4: 0 meant no resistance, while 4 indicated stiffness of the limb in flexion or extension [40]. In 1987, Bohannon and Smith modified the Ashworth scale by adding a score of 1+ to increase the test’s sensitivity [41]. The range of motion of the radiocarpal joint was measured using a goniometer.

The motor function of the upper limb after a stroke was assessed using the Fugl–Meyer Assessment of Motor Recovery. The FMA-UE is frequently employed for evaluating sensory–motor dysfunction in the upper limb following a stroke. It is widely used around the world; is practical for use in clinical settings; and is highly valid, reliable and responsive. It is commonly used to evaluate the severity of a stroke and quantify recovery. Furthermore, the FMA-UE demonstrates high intra- and inter-rater reliability, with reported values exceeding 0.90 in both the chronic and subacute phases [42,43].

Functional independence and autonomy were assessed using the modified Katz Activities of Daily Living (ADL) scale. This scale covers routine physical activities such as eating, dressing, bathing, mobility, using the toilet, and maintaining personal hygiene [44]. Assessing an individual’s ability to perform activities of daily living is crucial for determining their functional independence, overall health, and potential care needs, particularly in cases of significant paresis and functional impairment. The Visual Analog Scale (VAS) was used to evaluate pain intensity in the wrist flexors, as well as pain associated with the rESWT procedure. Pain during the procedure was reported using the VAS, and adverse events following the procedure were reported. The range of motion in the radiocarpal joint (extension) was analyzed due to the prevalence of a flexion pattern in patients with spasticity following a stroke.

## 3. Results

A statistical analysis was conducted using Statistica 13.3 software. The ESWT group and non-ESWT group were compared by the distribution of nominal variables using Pearson’s chi-square test with Yates’ correction or Fisher’s exact test when required due to small subgroup sizes. The normality of the distributions of continuous variables was tested using the Shapiro–Wilk test. To determine whether results on individual scales had changed over time, post hoc analysis was performed using the nonparametric Friedman ANOVA test and Mann–Whitney U test. In accordance with Bonferroni’s correction, the test probability (*p*) was multiplied by the number of comparisons for the post hoc tests. Spearman’s correlation coefficient was calculated to examine the relationship between results on individual scales, as well as changes on individual scales. No significant differences in baseline demographic characteristics were found between the groups.

A total of 62 patients who had suffered a stroke were included in the study. In the early phase, up to 6 months after stroke, spasticity of ≥2 on the MAS in the upper limb was observed in 20 patients in the ESWT group and in 11 patients in the non-ESWT group. The chronic phase was observed in 31 patients, including 22 from the ESWT group and 9 from the non-ESWT group. The shortest time since stroke was 1 month and was present in both groups, while the longest was 296 months (over 24 years) in the ESWT group and 265 months (over 22 years) in the non-ESWT group. Right-sided spasticity predominated in both groups. In the ESWT group, this affected 55 percent of participants (23 patients), while 45 percent (19 patients) presented with spasticity on the left side. In the non-ESWT group, the percentage of patients with right-sided involvement was higher at 70 percent (14 patients), while left-sided spasticity occurred in 30 percent (6 patients). Additional details are provided in Table 1.

The ESWT group showed improvements in upper-limb motor function (FMA-UE, *p* < 0.0001) and functional independence (ADL, *p* < 0.0001). They also experienced an increase in radiocarpal joint range of motion (*p* > 0.0001) and a reduction in spasticity (MAS, *p* < 0.0001) (Table 2). The results obtained one week after the procedure in the ESWT group showed improvement compared to baseline values. Scores on the FMA-UE, ADL, and ROM scales increased. A significant improvement in upper-limb motor function was observed in the ESWT group, with the mean FMA-UE score increasing by 22.88 points (from 58.17 in the first week to 81.05 in the fifth week). Meanwhile, functional independence increased from 1.38 to 3.64, representing a 164-percent improvement. In addition, an increase in range of motion was noticed, with average values rising from 24.43 to 42.43. There was a reduction in spasticity, a decrease in MAS scores, and a reduction in pain on the VAS. After five weeks in the ESWT group, a statistically significant improvement in outcomes was observed (*p* < 0.05) compared with the results obtained in the first week following ESWT. Pain scores on the VAS for the wrist and on the VAS for ESWT showed no significant changes after one week (*p* > 0.05). Patients rated wrist pain without ESWT at an average of 1.95, which decreased to 1.02 by week 5. Pain during the ESWT procedure averaged 2.81 and decreased to 1.95. However, after five weeks, a reduction in pain was observed compared to baseline (*p* = 0.0200 for the VAS for ESWT and *p* = 0.0268 for the VAS for the wrist).

Among patients in the non-ESWT group, motor function, measured by the FMA-UE scale, increased moderately from 61.4 (11.4) to 65.0 (9.9) after one week of the rehabilitation program. After 5 weeks, the value increased further to 70.8 (10.7) (*p* < 0.0001) compared with the second assessment. However, no pain reduction estimated by VAS was observed after 5 weeks (*p* = 0.4966). Spasticity, as measured by the MAS, decreased slightly from 2.9 (0.4) at baseline to 2.7 (0.6), remaining at this level after five weeks of rehabilitation. The changes in parameters were minor (*p* = 0.0724). Patients’ self-care abilities improved as estimated by the ADL scale from 1.5 (1.7) to 1.7 (1.7) after the first week. After five weeks of the rehabilitation program, we observed a significant increase of 2.4 (1.8) (*p* = 0.0009). In the non-ESWT group, there was a slight (*p* > 0.05) increase in radiocarpal joint extension range from 25.9° (6.2) to 26.1° (5.0) at the second assessment and to 28° (7.0) at the third assessment. Due to improvements in hand motor function (FMA-UE) and functional independence (ADL) in both groups, we examined whether the therapeutic effect was more clearly marked in the ESWT group (Table 3).

The baseline mean scores on these scales did not differ significantly between the two groups (*p* = 0.4205 for FMA and *p* = 0.8625 for ADL). However, after 5 weeks, the results were significantly higher (*p* < 0.05) in the ESWT group (*p* = 0.0080 for FMA and *p* = 0.0124 for ADL). To assess whether a reduction in spasticity is associated with a reduction in pain, we calculated the correlation coefficient for the Modified Ashworth Scale (MAS), Visual Analog Scale (VAS), and radiocarpal joint range (ROM).

**Figure 4:**
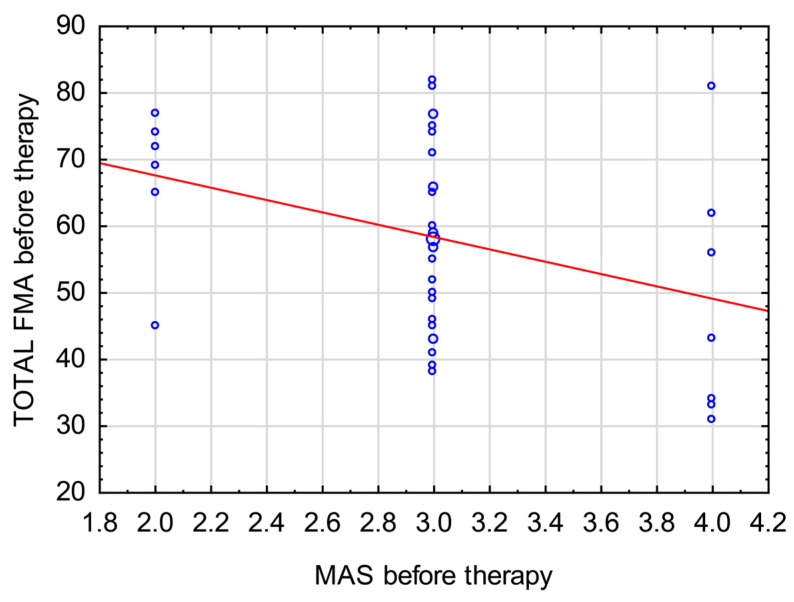
Analysis of FMA-UE and MAS correlation results prior to the commencement of therapy.

**Figure 5:**
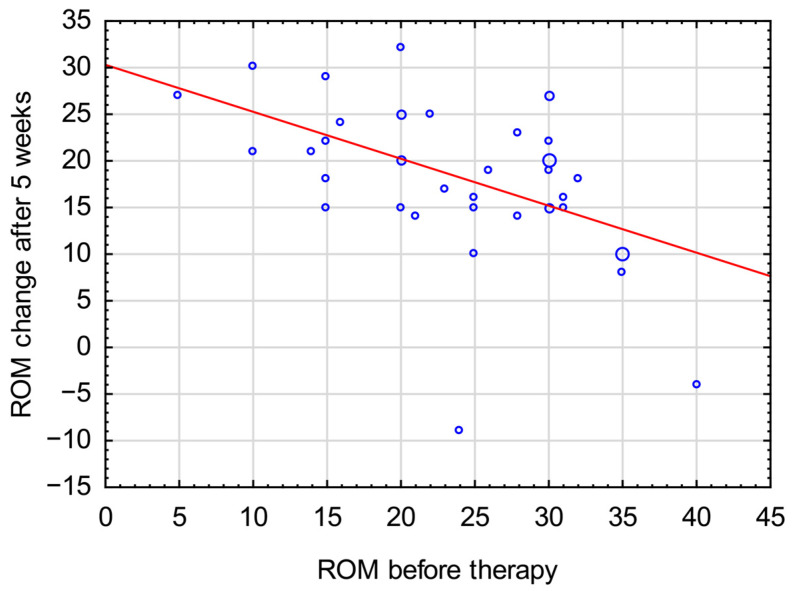
Analysis of the results of the correlation between radiocarpal joint extension range before therapy and after 5 weeks.

Patients with higher levels of spasticity according to MAS achieved lower average scores in terms of motor function improvement based on FMA-UE (r = −0.35, *p* = 0.0238). Improvement in spasticity reduction is associated with increased upper-limb mobility (Figure 4). It was observed that patients who initially had low radiocarpal joint extension ROM achieved significantly greater improvement in ROM after 5 weeks (r = −0.52, *p* = 0.0003) (Figure 5). However, no correlation was found between the decrease in pain on the VAS and the change in the radiocarpal joint extension range score (r = −0.13, *p* = 0.4169).

The results indicate that shockwave therapy is more beneficial for patients with greater ROM deterioration at baseline. Analysis of the results showed no correlation between the level of spasticity according to MAS and the self-care results achieved on the ADL scale. It is imperative to acknowledge that the functional independence score serves as a comprehensive evaluation of activities of daily living. This encompasses not only manual tasks but also activities such as transferring, toileting, and maintaining continence. Analysis of functional improvements in ADL revealed marked progress in the ESWT group, particularly in tasks requiring the use of the affected hand. The ability to dress using the affected limb was possible in 5 patients in week 1, while in week 5, this number increased to 33. In the non-ESWT group, an increase in this ability was noted from 4 to 9 patients. Regarding toilet use, the ESWT group showed improvement from 11 patients in week 1 to 15 in week 5, while no change was recorded in the non-ESWT group. The ability to eat independently improved in the ESWT group from 7 to 36 patients, while in the non-ESWT group, this number increased by 3 patients. Regarding personal hygiene, a significant increase in the number of patients was noted; in the ESWT group, this ability was present in 9 patients in week 1 and in 28 patients in week 5. In the non-ESWT group, the improvement was smaller and included only 2 additional patients by week 5 of the study. Spearman’s correlation analysis was conducted on the non-ESWT and ESWT groups combined and separately, at baseline and at two time points. This revealed associations between the patients’ clinical status (subacute/chronic), their treatment group and stage of therapy, and the results of the functional scales.

No significant differences were found between the group of patients who underwent ESWT and the group that did not undergo this therapy prior to the start of the study; therefore, baseline correlations were calculated for both groups combined. Among patients in the chronic phase, a very strong positive correlation was observed between the FMA-UE and ADL scores (r = 0.89; *p* = 0.0013), corresponding to a very large effect size (r^2^ = 0.79). This indicates that better upper-limb function was associated with greater independence in performing activities of daily living. Furthermore, a strong positive correlation was observed between the range of motion for extension at the wrist-radius joint and the ADL scale scores (r = 0.77; *p* = 0.0155), also corresponding to a large effect size (r^2^ = 0.59), suggesting that a greater range of motion was associated with a higher level of patient functioning. In many analyses, a strong negative correlation was demonstrated between the severity of spasticity assessed using the MAS and the range of motion in wrist extension. The strongest correlation was observed in patients in the subacute phase after one week of observation in the group that did not undergo ESWT (r = −0.71; *p* = 0.0149), which corresponds to a large effect size (r^2^ = 0.50). A positive correlation was also demonstrated between upper-limb function and the range of motion in wrist extension, with the strongest correlation observed after 5 weeks in the group not treated with ESWT during the chronic phase (r = 0.75; *p* = 0.0202; r^2^ = 0.56). Despite the observed relationships between upper-limb function, functional independence, spasticity, and range of motion of the joints, no significant correlations were found with pain intensity assessed on the VAS. The obtained correlation coefficients indicated small or moderate effect sizes that did not reach statistical significance. Detailed results of all correlation analyses are presented in the Supplementary Material.

## 4. Discussion

The results provide new insights into the potential application of rESWT to the flexor muscles of the wrist. According to Hefter’s classification, wrist flexion occurs in three of the five upper-limb spasticity patterns, making it one of the most common distal manifestations of upper-limb spasticity following a stroke. A study by Doussoulin et al. (2020), which examined 136 patients, found that the prevalence of spasticity in the wrist joint increases between 10 days and 3 months after stroke onset and remains stable for up to 12 months or longer [45]. Given the high prevalence of spasticity in the wrist joint, our study focused specifically on this area to assess the efficacy of extracorporeal shockwave therapy (ESWT). Our research results indicated a reduction in spasticity; an increase in radiocarpal joint extension ROM; improved independence in activities such as eating, dressing, and bathing; and improved hand motor function in stroke patients following rESWT application. Previous studies suggested the effectiveness of ESWT in reducing upper-limb spasticity [37,46]. However, there has been a lack of studies evaluating the patient’s pain experience during the rESWT procedure [47]. During ESWT, some patients reported mild pain, with an average intensity of 2.40 points on the VAS during the second session and 1.95 points during the fourth. All patients experienced transient skin redness that resolved spontaneously within 24 h. No serious adverse events were reported. The decrease in pain between the second and fourth sessions may indicate gradual adaptation to the therapy. Due to the potential for pain during rESWT, both procedure-related pain and hand pain were monitored. This study is one of the first comprehensive analyses to evaluate the impact of ESWT on pain, spasticity, hand function, and functional independence, providing data relevant to clinical practice.

Improvement and statistical significance were recorded in all scales and parameters in the ESWT group (*p* < 0.05), except for VAS. As the range of motion in the radiocarpal joint improved, a parallel improvement in hand function as assessed by the FMA-UE was observed, along with an increase in functional independence in activities of daily living (ADL) requiring the use of the upper limb. The ADL scale was used to assess independence, as it is a classic and well-established tool for evaluating basic activities of daily living. The ADL scale is a tool widely used in Polish clinical practice, particularly in geriatrics, long-term care, and the functional assessment of hospitalized patients, including those who have suffered a stroke [48,49]. However, the limitations associated with the use of this tool must be taken into account. The limited sensitivity of the scale and the possibility of a ceiling effect may make it difficult to detect subtle functional changes. In future studies, it seems reasonable to use additional, more detailed functional assessment tools, such as the Functional Independence Measure (FIM), the Barthel Index, or the Disability Assessment Scale (DAS), which could enable a more comprehensive evaluation of the effects of rehabilitation [35,38,50]. ESWT induces significant and beneficial changes to the rheological properties of muscle tissue, primarily by increasing elasticity and improving muscle tone. Acting as a mechanical stimulus, ESWT releases muscle restrictions and promotes tissue remodeling, making it an effective, noninvasive method for improving muscle function. This makes it particularly useful in sports medicine and, increasingly, in the treatment of spasticity [47].

Contractures are associated with poorer functional outcomes and hinder the rehabilitation process [51]. Beebe et al. (2009) [52] demonstrated that measuring active range of motion one month after stroke onset can predict 71 percent of the variance in upper-limb function three months later. A greater deficit in wrist range of motion one month after stroke strongly correlates with poorer upper-limb functional outcomes three months later (*p* > 0.01) [52]. In our study, patients in the ESWT group, who initially exhibited greater restriction of wrist extension, achieved the greatest improvement, by an average of 19 degrees, corresponding to a 78-percent increase. ESWT was particularly effective in patients with limited wrist extension. Although no differences were observed between the groups before treatment or one week after treatment, after 5 weeks, the ESWT group achieved significantly better FMA-UE and ADL scores.

The analysis showed that greater spasticity prior to therapy was associated with poorer upper-limb function as assessed on the FMA-UE scale. In the ESWT group, a gradual reduction in spasticity was observed after 1 and 5 weeks of therapy. The strongest correlations occurred in the chronic phase, where a strong negative correlation was found between the MAS and range of motion after 1 week (r = −0.67; *p* = 0.0006) and after 5 weeks of therapy (r = −0.64; *p* = 0.0014). The results indicate that a reduction in spasticity promotes improved upper-limb mobility after a stroke, particularly in the chronic phase, a finding also confirmed by Brunelli et al. (2022) [34]. A key finding of our study was that patients with the greatest initial limitation of wrist extension achieved the greatest improvement in ROM. A similar relationship was also observed by Savevska et al. (2016), whose case report of a female patient in the chronic phase of a stroke indicates that significant baseline functional deficits may be associated with exceptionally large improvements following treatment [38]. In the study by Nada et al. (2024), it was shown that patients with the most advanced structural muscle changes (grade IV on the Heckmatt scale), in whom muscle tissue had been largely replaced by fat and fibrosis, responded significantly less well to ESWT in the long term than those with muscle tissue not replaced by fat and fibrosis [53,54]. The study employed four ESWT sessions, during which 1500 pulses were delivered at a pressure of 2.5 bar and a frequency of 4 Hz. These results suggest that while more severe clinical symptoms may be associated with greater potential for improvement, advanced structural changes in the muscle represent a significant limitation to the effectiveness of treatment.

The negative correlation between MAS and FMA-UE, which was strongest in the chronic phase of stroke after 5 weeks of ESWT (r = −0.57; *p* = 0.0058), indicates a relationship between reduced spasticity and improved upper-limb function. This is confirmed by higher FMA-UE scores in the ESWT group than in the control group (81.0 ± 14.6 vs. 70.8 ± 10.7; *p* = 0.0080). Similar effects were described by Tabra et al. (2021) when using parameters of 15 Hz, 2.8 bar, and 2000–3000 pulses [55].

A strong positive correlation was observed between FMA-UE scores and ADL scores, indicating that improved upper-limb motor function was associated with greater independence in patients. This relationship was observed in both the subacute and chronic phases of stroke, with the strongest correlation observed in the chronic phase. These results are consistent with the findings of Özbek et al. (2025) [35], who demonstrated that rESWT improves both upper-limb function and functional independence. The similarity in results may stem from the comparable therapy parameters used in both studies [35].

The VAS ESWT and VAS wrist did not change significantly after one week; however, a significant reduction in pain was observed after 5 weeks (*p* = 0.0200; *p* = 0.0268). No significant correlations were found between pain intensity and spasticity, however. Similar results were obtained by Wong et al. (2023), who demonstrated that pain reduction following botulinum toxin treatment was not associated with the degree of spasticity reduction [56]. Similarly, Lynning et al. (2020) suggest that pain is more strongly associated with the subjective perception of spasticity, general hypertonia, and disease severity than with objectively assessed spasticity [57]. Stronger correlations between spasticity, upper-limb function, and range of motion in patients in the chronic phase of stroke suggest that the impact of spasticity on motor function was more pronounced in this group than in the subacute phase. This means that a reduction in spasticity was more strongly associated with improved upper-limb function and increased ROM. However, no significant correlations were found between pain intensity and spasticity or range of motion, indicating that post-stroke pain also depends on other factors, such as centrally generated pain, secondary musculotendinous changes, limited joint mobility, or coexisting sensory disturbances. Furthermore, the lack of a relationship between changes in pain and ROM suggests that functional improvement resulted primarily from reduced spasticity and improved tissue properties, rather than from an analgesic effect. Nevertheless, after 5 weeks of ESWT, a significant reduction in pain was observed compared to baseline values (VAS: *p* = 0.0200; VAS for the wrist: *p* = 0.0268), which may indicate a partially independent analgesic effect of the therapy. Similar results were obtained by Li et al. (2020) [11], who observed a reduction in pain and spasticity in patients with chronic stroke after five rESWT sessions. The authors noted that the analgesic effect may result from a reduction in inflammatory processes, decreased nociceptor excitability, and improved biomechanical properties of tissues. This suggests that the analgesic effect of rESWT does not depend solely on the reduction in spasticity [11].

Pain in stroke patients can have various causes, ranging from muscle spasticity and joint stiffness to secondary musculoskeletal complications [58]. Spasticity correlates with a higher risk of pain after a stroke [59]. ESWT therapy may reduce pain by stimulating the secretion of endogenous analgesic substances, modulating pain conduction pathways, or reducing local inflammation [60]. Troncati et al. (2013) [61] did not demonstrate a significant improvement in pain as assessed by the VAS following rESWT, although they did note a reduction in spasticity and an improvement in upper-limb motor function. Changes in pain intensity did not correlate with improvements in clinical parameters, confirming that pain reduction following a stroke may occur more slowly than functional improvement [61].

In our study, the ESWT intervention took place weekly for a period of four weeks, with observation and evaluation taking place before the start of the study, after the initial week of treatment, and one week after the fourth ESWT intervention. At the same time, patients received medical, psychological and speech therapy, as well as conventional rehabilitation care at the neurological rehabilitation ward. Among the 168 rESWT procedures performed, only 12 of these did not cause pain for the patient. 90.4 percent of patients experienced slight discomfort during the procedure (VAS > 1).

Analysis of the results in relation to the previously defined research hypotheses confirmed the main hypothesis (H1) regarding the effect of rESWT on spasticity reduction. Secondary hypotheses H2 and H3, relating to improvements in upper-limb motor function and increased patient independence in activities of daily living, were also confirmed. The results confirming hypotheses H2 and H3 remained statistically significant even after accounting for multiple comparisons. However, no evidence was found to support hypothesis H4, which posited a significant reduction in pain. Despite the absence of significant differences between the groups, a gradual decrease in pain intensity was observed in the ESWT group during the follow-up period. This may suggest a potential analgesic effect of the therapy, which could be demonstrated with greater statistical power in a study with a larger sample size or a longer follow-up period. Due to the analysis of several dependent variables, we considered the possibility of Type I error accumulation resulting from multiple comparisons, a phenomenon described extensively in the methodological literature on clinical trials [62]. Each analyzed variable was treated as a separate endpoint corresponding to a previously defined research hypothesis. Furthermore, the *p*-values obtained for results supporting the hypotheses were significantly lower than the adopted significance level (*p* < 0.0001), which indicates a high degree of stability in the results and suggests that the study’s main conclusions remain robust against the potential impact of multiple testing.

Our study has certain limitations. Firstly, the study protocol included only four shockwave treatments, with three observation points for the patients. Patients were examined for the final time after 5 weeks of hospitalization. This approach does not allow for the observation of long-term effects of ESWT therapy. Future studies should therefore include additional time points covering at least 3, 6, and 12 months, as in the study by Guo et al. (2019) [37]. To enhance the accessibility of ESWT therapy for patients suffering from post-stroke spasticity, further research is necessary. Such research should involve a substantial number of participants. Conducting future studies on larger groups of patients would strengthen the reliability of the results, improve the quality of the data, and enhance their statistical value. Another limitation of the study was the heterogeneity in disease duration among the participants. Although there were no significant differences between the groups, the varying duration of the disease may have influenced individual responses to treatment and constituted a potential source of variability in the results obtained. Another limitation worth mentioning is the lack of blinding of the participants. Due to the nature of the ESWT intervention used, participants were aware of their assignment to either the treatment or control group, which may have influenced their subjective assessment of the therapy’s effects. Therefore, the results obtained should be interpreted with consideration of the potential risk of bias. Another limitation of the study is its single-center design, which may limit the generalizability of the results to a broader population of stroke patients with spasticity. The non-ESWT and ESWT groups were unequal in size, which resulted from the adopted randomization ratio and may have affected the statistical power and comparability of the groups. The lack of blinding and the absence of allocation concealment procedures may have increased the risk of systematic errors. Future multicenter studies involving larger research teams, the use of allocation concealment procedures, and assessment by independent, blinded assessors may contribute to improving methodological quality and the level of scientific evidence.

Despite multiple studies examining the use of ESWT in reducing spasticity, there is still no uniform treatment protocol for specific conditions. The parameters currently in use vary between authors and centers, making it difficult to compare and implement the method in clinical practice. Future studies on the use of ESWT to reduce spasticity in stroke patients should focus on developing a transparent protocol that defines the optimal procedure parameters and criteria for its use, with the aim of increasing its effectiveness and repeatability.

## 5. Conclusions

In the present study, four sessions of radial extracorporeal shockwave therapy (rESWT) targeting the wrist flexor muscle group (flexor carpi radialis, flexor carpi ulnaris, palmaris longus, pronator teres, pronator quadratus, and flexor digitorum superficialis), combined with conventional rehabilitation, were associated with short-term improvements in upper-limb motor function, reduced spasticity, increased radiocarpal joint extension, and greater functional independence in stroke patients with upper-limb spasticity (MAS ≥ 2). The results suggest that improvements in upper-limb function and reductions in spasticity may occur independently of changes in pain perception. However, due to the short follow-up period and methodological limitations of the study, including its single-center design, further large-scale studies with long-term follow-up are needed to confirm the durability of these effects and establish their long-term clinical significance.

## Figures and Tables

**Table 1:** Characteristics of study participants.

Clinical Data	Overall Patients (*N* = 62)	ESWT Group (*N* = 42)	Non-ESWT Group (*N* = 20)	*p*
State Chronic > 6 months Subacute ≤ 6 months	42 (68%) 20 (32%)	22 (52%) 20 (48%)	9 (45%) 11 (55%)	0.7859
Gender Women Men	19 (31%) 43 (69%)	10 (24%) 32 (76%)	9 (45%) 11 (55%)	0.1624
Type of stroke Ischemic Hemorrhagic	57 (92%) 5 (8%)	39 (93%) 3 (7%)	18 (90%) 2 (10%)	0.5223
Brain hemisphere Left Right	42 (68%) 20 (32%)	23 (55%) 14 (70%)	19 (45%) 6 (30%)	0.3862
Number of strokes 1 2 3+	51 (82%) 8 (13%) 3 (5%)	35 (83%) 5 (12%) 2 (5%)	16 (80%) 3 (15%) 1 (5%)	0.8652
Nicotinism Yes No	47 (76%) 12 (19%)	35 (83%) 7 (17%)	12 (71%) 5 (29%)	0.4566
Hand spasticity Right Left	37 (60%) 25 (40%)	23 (55%) 19 (45%)	14 (70%) 6 (30%)	0.3862

**Table 2:** Comparison of results in the ESWT group and the control group after 1 and 5 weeks of follow-up.

Research Instruments	Baseline Mean ± SD		1-Week Follow-Up Mean ± SD		5-Week Follow-Up Mean ± SD		*p*	
	ESWT group (*N* = 42)	non-ESWT group (*N* = 20)	ESWT group (*N* = 42)	non-ESWT group (*N* = 20)	ESWT group (*N* = 42)	non-ESWT group (*N* = 20)	ESWT group (*N* = 42)	non-ESWT group (*N* = 20)
FMA-UE	58.2 (14.5)	61.4 (11.4)	65.1 (15.1)	65.0 (9.9)	81 (14.6)	70.8 (10.7)	<0.0001	<0.0001
MAS	3.0 (0.6)	2.9 (0.4)	2.4 (0.7)	2.7 (0.6)	1.6 (0.7)	2.7 (0.7)	<0.0001	0.0724
ADL	1.4 (1.4)	1.5 (1.7)	1.7 (1.6)	1.7 (1.7)	3.6 (1.4)	2.4 (1.8)	<0.0001	0.0009
VAS ESWT	2.8 (2.6)	---	2.4 (2.5)	---	2.0 (2.1)	---	0.0220	-
VAS wrist	1.9 (2.5)	3.6 (2.7)	1.7 (2.2)	3.2 (2.8)	1.0 (1.9)	3.7 (2.8)	0.0116	0.4966
Extension radiocarpal joint [°]	24.4 (8)	25.9 (6.2)	34.7 (8.7)	26.1 (5.0)	42.4 (8)	28 (7.0)	<0.0001	0.4677

Post hoc test results: FMA-UE: before vs. 1 week after, *p* < 0.0001; 1 week after vs. 5 weeks after, *p* < 0.0001; MAS: before vs. 1 w.a., *p* < 0.0001; 1 w.a. vs. 5 w.a., *p* < 0.0001; ADL before vs. 1 w.a., *p* < 0.0001; 1 w.a. vs. 5 w.a., *p* < 0.0001; VAS ESWT before vs. 1 w.a., *p* < 0.0001; 1 w.a. vs. 5 w.a., *p* < 0.0001; VAS wrist before vs. 1 w.a., *p* < 0.0001; 1 w.a. vs. 5 w.a., *p* < 0.0001; extension radiocarpal joint before vs. 1 w.a., *p* < 0.0001; 1 w.a. vs. 5 w.a., *p* < 0.0001.

**Table 3:** Changes in functional status estimations over time in the ESWT and non-ESWT groups.

Research Instruments	ESWT Group (*N* = 42)	Non-ESWT Group (*N* = 20)	*p*
FMA-UE baseline	58.2 (14.5)	61.4 (11.4)	0.4205
FMA-UE 1-week follow-up	65.1 (15.1)	65.0 (9.9)	0.9940
FMA-UE 5-week follow-up	81.0 (14.6)	70.8 (10.7)	0.0080
ADL baseline	1.4 (1.4)	1.5 (1.7)	0.8625
ADL 1-week follow-up	1.7 (1.6)	1.7 (1.7)	0.7864
ADL 5-week follow-up	3.6 (1.4)	2.4 (1.8)	0.0124

## Data Availability

The original contributions presented in this study are included in the article. In addition, as Supplementary Material, a table containing the results of correlation analyses between the study groups and all analyzed variables has been made available. Further questions regarding the data may be directed to the corresponding author.

## Supplementary Materials

The following supporting information can be downloaded at https://www.mdpi.com/article/10.3390/jcm15155879/s1, Table S1. Comparison of correlations among clinical assessment scales in patients in the subacute and chronic stages following stroke.

## Abbreviations

FMA-UE: Fugl–Meyer Assessment of Motor Recovery after Stroke; MAS: Modified Ashworth Scale; ADL: Modified Katz Activities of Daily Living Scale; VAS: Visual Analog Scale; ROM: range of motion; ESWT: extracorporeal shockwave therapy; rESWT: radial shockwave therapy.
