# Supplementary material for: The Role of Radial Shockwave Therapy in Reducing Spasticity and Improving Upper-Limb Function Following Stroke
Source: J Clin Med. 2026 Jul 28;15(15):5879. doi: 10.3390/jcm15155879 (PMC13467381; doi:10.3390/jcm15155879)
Supplement: Supplementary file 1 [file jcm-15-05879-s001.zip › jcm-4406507-supplementary.pdf]

Supplementary Table S1. Comparison of correlations among clinical assessment scales in patients in the subacute and chronic stages following stroke.

**Baseline non-ESWT and ESWT groups**				
	Chronic stage (n=31)		Subacute stage (n=31)	
	Spearman's correlation coefficient R	p	Spearman's correlation coefficient R	p
FMAUE vs MAS	-0.29	0.1186	-0.42	0.0197
FMAUE vs ADL	0.48	0.0062	0.37	0.0399
FMAUE vs ROM	0.26	0.1567	0.13	0.5020
MAS vs ADL	-0.20	0.2760	-0.43	0.0147
MAS vs ROM	-0.46	0.0085	-0.32	0.0805
ADL vs ROM	0.41	0.0205	0.13	0.4704
**ESWT group 1 week follow-up**				
	Chronic stage (n=22)		Subacute stage (n=20)	
	Spearman's correlation coefficient R	p	Spearman's correlation coefficient R	p
FMAUE vs MAS	-0.36	0.1044	-0.36	0.1239
FMAUE vs ADL	0.23	0.3121	0.57	0.0092
FMAUE vs ROM	0.44	0.0414	0.54	0.0140
MAS vs ADL	-0.10	0.6603	-0.32	0.1719
MAS vs ROM	-0.67	0.0006	-0.02	0.9182
ADL vs ROM	0.12	0.5912	0.28	0.2240
**non-ESWT group 1 week follow - up**				
	Chronic Stage (n=9)		Subacute stage (n=11)	
	Spearman's correlation coefficient R	p	Spearman's correlation coefficient R	p
FMAUE vs MAS	-0.23	0.5531	-0.17	0.6195
FMAUE vs ADL	0.84	0.0044	0.19	0.5804
FMAUE vs ROM	-0.25	0.5165	0.39	0.2330
MAS vs ADL	-0.42	0.2609	-0.32	0.3353
MAS vs ROM	0.09	0.8130	-0.71	0.0149
ADL vs ROM	-0.41	0.2682	0.35	0.2973
**ESWT group 5 weeks follow – up**				
	Chronic stage (n=22)		Subacute stage (n=20)	
	Spearman's correlation coefficient R	p	Spearman's correlation coefficient R	p
FMAUE vs MAS	-0.57	0.0058	-0.14	0.5426
FMAUE vs ADL	0.51	0.0142	0.78	0.0001
FMAUE vs ROM	0.57	0.0055	0.45	0.0485
MAS vs ADL	-0.48	0.0227	-0.04	0.8613
MAS vs ROM	-0.64	0.0014	-0.47	0.0371
ADL vs ROM	0.35	0.1108	0.33	0.1530
**non-ESWT group 5 weeks follow - up**				
	Chronic stage (n=9)		Subacute stage (n=11)	
	Spearman's correlation coefficient R	p	Spearman's correlation coefficient R	p
FMAUE vs MAS	-0.36	0.3444	-0.30	0.3676
FMAUE vs ADL	0.89	0.0013	0.26	0.4340
FMAUE vs ROM	0.75	0.0202	0.54	0.0838
MAS vs ADL	-0.58	0.1024	-0.34	0.3030
MAS vs ROM	-0.41	0.2701	-0.14	0.6756

ADL vs ROM	0.77	0.0155	-0.24	0.4838
